# On the Structure and Source of Individual Differences in Toddlers' Comprehension of Transitive Sentences

**DOI:** 10.3389/fpsyg.2021.661022

**Published:** 2021-10-20

**Authors:** Seamus Donnelly, Evan Kidd

**Affiliations:** ^1^Research School of Psychology, The Australian National University, Canberra, ACT, Australia; ^2^Australian Research Council Center of Excellence for Dynamics of Language, Canberra, ACT, Australia; ^3^Language Development Department, Max Planck Institute for Psycholinguistics, Nijmegen, Netherlands; ^4^Donders Institute for Brain, Cognition and Behavior, Radboud University, Nijmegen, Netherlands

**Keywords:** grammar, individual differences, intermodal preferential looking paradigm, mixture models, usage-based account of language, early abstraction account of language

## Abstract

How children learn grammar is one of the most fundamental questions in cognitive science. Two theoretical accounts, namely, the Early Abstraction and Usage-Based accounts, propose competing answers to this question. To compare the predictions of these accounts, we tested the comprehension of 92 24-month old children of transitive sentences with novel verbs (e.g., “The boy is gorping the girl!”) with the Intermodal Preferential Looking (IMPL) task. We found very little evidence that children looked to the target video at above-chance levels. Using mixed and mixture models, we tested the predictions the two accounts make about: (i) the structure of individual differences in the IMPL task and (ii) the relationship between vocabulary knowledge, lexical processing, and performance in the IMPL task. However, the results did not strongly support either of the two accounts. The implications for theories on language acquisition and for tasks developed for examining individual differences are discussed.

## Introduction

The emergence of grammatical knowledge marks a significant watershed in the language development of a child. Without explicit instruction, children rapidly learn the intricate language-specific conventions for mapping grammatical forms to meanings. For example, English-speaking children must learn that for transitive sentences such as *the dog is chasing the cat*, the SUBJECT refers to the agent and the OBJECT refers to the patient, but for passive sentences such as *the cat is being chased by the dog*, the SUBJECT refers to the patient and the OBJECT refers to the agent.

The explanation of this process is highly contested, owing to it being a key battleground in debates regarding the innateness of linguistic knowledge (Ambridge and Lieven, 2011). Broadly speaking, there are two classes of explanations for how children acquire grammar, which make different assumptions about how and what children learn. Usage-Based theories assume that children rely on domain-general cognitive processes, such as pattern recognition and statistical learning, to learn grammatical constructions in much the same way as they learn words (Bates and MacWhinney, 1982; Tomasello, 2003; Ambridge and Lieven, 2015). In particular, Usage-Based theories assume that children build a grammatical system based on initially concrete, lexically based knowledge. Accordingly, the early grammatical knowledge of children does not consist of abstract mappings between concepts like SUBJECT and agent but is instead tied to particular concrete lexical items. For instance, children's knowledge of the verb kick (and similar action verbs) is described by an initially low-scope kickee-KICK-kicker formula. Across early development, and specifically, as children acquire more verbs, they construct more and more abstract representations, which eventually approximate linguistic categories such as noun, verb, subject, object, agent, and patient. Usage-Based accounts make two key predictions about the developing grammatical knowledge of children: first, because their earliest sentences are highly concrete, children should be conservative in generalizing grammatical constructions to novel verbs; second, because grammatical knowledge is lexically anchored, there should be a tight relationship between measures of vocabulary and grammatical proficiency (Marchman and Bates, 1994).

Unlike Usage-Based accounts, Early Abstraction accounts assume that children have early access to abstract linguistic categories, such as nouns, verbs, subjects, objects, agents, and patients, and possibly biases for linking syntactic and thematic structures (Lidz et al., 2003; Fisher et al., 2020). Such knowledge may be innate or the product of early pre-linguistic experience. Either way, the role of learning is to determine which words belong to which syntactic categories and how these categories are combined to create an inventory of constructions within a specific language (Valian, 2014; Lidz and Gagliardi, 2015; Fisher et al., 2020). Because children have access to these categories early in development, they are assumed to represent early sentences in an abstract format. For example, children who have begun producing a sentence such as *the boy is feeding the girl* are assumed to be representing it using syntactic categories, such as NOUN-VERB-NOUN, and semantic categories, such as agent-action-patient. Therefore, these accounts predict that, once a child can produce a given grammatical construction, they will readily generalize it to novel verbs (Valian, 2014; Messenger and Fisher, 2018; Fisher et al., 2020). Any failure to do so, according to this account, will likely reflect processing constraints or a lack of semantic knowledge of the verb, separate from the knowledge children have of syntax (Naigles, 2002; See Fisher, 2002). Moreover, Early Abstraction accounts assume that, while increased lexical knowledge may help children determine how these linguistic categories are configured within particular constructions, it plays no role in strengthening these abstract representations. As such, these accounts predict that, once children begin using a construction with a specific verb, further lexical knowledge will not play a role in strengthening the abstract representation of that construction (Messenger and Fisher, 2018).

Given these different predictions, the two accounts can be disentangled by examining the ability of children to use novel verbs in known grammatical constructions. A classic paradigm for examining this involves training a novel verb in one grammatical construction (e.g., an intransitive) and eliciting a transitive sentence with the same verb. In general, these studies find that young children (e.g., 2; 0) are quite conservative about generalizing from one construction to another, but that children generalize more readily with age (Tomasello, 2000; Ambridge and Lieven, 2011). While these results seem to suggest that the youngest children lack abstract linguistic categories and that their representations become increasingly abstract with age and linguistic experience, Fisher (2002) has noted that, given that most English verbs only occur in a subset of grammatical constructions, we should not expect children to assume a novel verb heard in one structure can be freely used in another. Therefore, the unwillingness of children to generalize the known construction to a novel verb may reflect a lack of evidence regarding the argument structure of the verb.

A more fruitful approach to testing these predictions is to see whether children can comprehend sentences with novel verbs (Fisher, 2002). If children can reliably interpret a sentence such as *the boy is gorping the girl* as referring to a causal scene in which a boy is acting on a girl, this suggests they are representing the transitive construction with abstract categories using their knowledge of grammar to infer the meaning of the verb (*via* syntactic bootstrapping, Gleitman, 1990). One method for examining this question is a version of the Intermodal Preferential Looking (IMPL) task, adapted by Gertner et al. (2006; see also Golinkoff et al., 1987; Naigles, 1990, 2021). In a set of four studies, Gertner et al. (2006) examined whether 21- and 25-month-old children could comprehend transitive sentences with nonce verbs. The participants saw two videos, each depicting a novel causal action with opposite participant roles, and heard a transitive sentence (e.g., *The boy is GORPING the girl*). The participants looked at the target video at above-chance levels in all four studies, suggesting that they had abstract knowledge of transitive argument structure. Moreover, in all the studies, the participants correctly interpreted the sentence within the first 2 s of the first trial. The authors noted that, because 21-month-old children, in particular, have very few verbs in their (productive) vocabularies, this finding strongly supports the Early Abstraction account. Similar results were reported by Ferndandes et al. (2006) and Noble et al. (2011) in samples of slightly older children (at least 27 months) using a forced-choice pointing paradigm.

However, Dittmar et al. (2008a) noted that the above-chance looks to transitive sentences could have been an artifact of the design. Prior to critical test trials, Gertner et al. (2006) included a set of familiarization trials in which children saw videos of two known actions and heard a target sentence, such as “the boy is washing the girl.” Because the familiarization trials included the same characters as the target trials, Dittmar et al. (2008a) argued that participants may have learned that, within the context of the task, sentences that start with “the boy” refer to actions where the male character is the agent. To test this possibility, the authors compared the performance of German-speaking 21-month-old children in the same paradigm under two conditions, with and without training. The with-training condition used the same familiarization procedure as Gertner et al., while the without-training condition used a modified familiarization procedure in which participants did not hear a transitive sentence (e.g., “This is called washing. Find washing!”). They found that the participants in the no-training condition did not look at the target at above-chance levels and that the children in the with-training condition looked at above-chance levels only in the final 2 s of the second trial (of two). The authors noted that the latter finding is qualitatively different from that of Gertner et al. (2006), who osberved above-chance looks to target in the earliest windows, and argued that the total pattern of results suggests that the children's representations of transitive sentences are likely quite fragile and tied to linguistic experience prior to testing.

This conclusion, however, is inconsistent with the findings reported by Scott et al. (2018). Across two studies, the second of which contained no training trials, they found that 21-month-old children reliably interpreted transitive sentences with novel verbs. Assuming comparability across testing procedures, this finding raises the possibility that the finding of Dittmar et al. (2008a) in the no-training condition reflects a language-based difference. One logical source of the difference could be the fact that German uses a case to mark participant roles, whereas English does so under only very limited circumstances (i.e., in pronouns). However, corpus studies show that word order is a highly reliable cue to thematic role assignment in German (Dittmar et al., 2008b), making this explanation unlikely. Another possibility is that Scott et al. (2018) used non-agentive subjects and non-causative actions (e.g., a ball jumping over a flower). However, if this were the locus of the difference, it would be difficult to reconcile with the findings of Gertner et al. (2006).

The totality of this evidence suggests that toddlers have representations of transitive sentences that are independent of the specific verbs used, although these representations may be quite fragile in nature, and there are notable discrepancies across studies in the existence, size, and timing of the effect (Ambridge and Lieven, 2015). It is not clear if these inconsistencies reflect differences in the samples used (the language of testing) or are artifacts of the relatively small sample sizes used in these studies (which are typically small and generally *N* ≤ 30). Therefore, there is a need for large sample replications of these studies.

However, even if 21-month-old children do reliably look at the target video in this version of the IMPL task, this result would not adjudicate between the Early Abstraction and Usage-Based accounts (Messenger and Fisher, 2018). The proponents of Early Abstraction theories could argue that because children know very few verbs by 21 months, the ability to generalize these structures to novel verbs suggests that children have acquired abstract syntactic categories (Gertner et al., 2006; Messenger and Fisher, 2018). However, the proponents of Usage-Based theories could respond by saying that, when presented with the task of using a novel linguistic stimulus to choose between two videos, very rudimentary representations of grammar are sufficient (Abbot-Smith and Tomasello, 2006; Ambridge and Lieven, 2015), an intuition supported by computational modeling showing that comprehension can be supported by much simpler representations than production with novel verbs (Chang et al., 2006).

Because neither of these accounts makes specific predictions about the age at which children should comprehend transitive sentences with novel verbs, the research strategy of examining whether children of a sufficiently young age can comprehend transitive sentences may not be viable (Ambridge and Lieven, 2015). An alternative approach is to leverage the fact that the two accounts make different assumptions about what is learned and, therefore, make different predictions about the structure of individual differences (Kidd et al., 2018a; Kidd and Donnelly, 2020). Recall that Early Abstraction accounts assume that children learn how to configure readily available abstract representations for specific constructions within their language. These accounts predict that, once children begin producing a particular grammatical construction, they will represent the structure in a sufficiently abstract format to immediately transfer it to new verbs [Messenger and Fisher, 2018; Fisher et al., 2020; see also Valian (2014) and Meylan et al. (2017) for a similar prediction about the use of determiners by children]. This suggests that, for a given construction, a sample of children will contain two groups of responders: those who have learned the construction and, therefore, look at the target at above-chance levels, and those who have not learned the construction and, therefore, look at the target at chance levels^1^. That is, participants will exhibit discrete individual differences. Note that this prediction of discrete individual differences refers to the form of variability in the children's knowledge of specific grammatical constructions. Early Abstraction accounts allow graded variability in the total number of constructions children have acquired, since it presumably takes more input to learn, for example, how English expresses the passive than how it expresses the active transitive. Moreover, such accounts allow children to vary in their processing of a given construction because of differences in non-syntactic variables, such as speech recognition and knowledge of relevant vocabulary. However, representations of the syntax of particular constructions should exhibit all-or-none variability.

On the other hand, Usage-Based accounts assume that children initially learn in an item-specific manner and gradually construct increasingly abstract representations. For example, in discussing the task-dependent success of children in syntactic productivity, Abbot-Smith and Tomasello (2006) argue that different tasks (preferential looking vs. production) likely require grammatical representations of different strengths. They propose that children can begin constructing abstract representations as soon as they have acquired multiple lexically specified argument constructions with sufficient semantic and functional overlap [see also, Ambridge and Lieven (2015) for a similar proposal]. Such representations may be sufficient for simple tasks, such as the IMPL, but not for the more challenging elicited production tasks. As these constructions become further entrenched, and the child learns more semantically and functionally similar pairs, their representation of relevant sentence structures will strengthen, up until a point where the child reaches an adult-like performance. Such an account is supported by the computational simulations of performance in the IMPL and a production task by Chang et al. (2006). Consistent with the arguments of Abbot-Smith and Tomasello (2006), the model required less input to complete the IMPL task than the production task. Moreover, for both tasks, the performance of the model improved with more input, up until it reached adult-like levels, as its grammatical representations strengthened with more input. Because Usage-Based models predict that the grammatical representations of children are input-driven, they, unlike Early Abstraction accounts, predict a pattern of graded individual differences in the knowledge children have of a given grammatical construction^2^.

These two predictions about the structure of individual differences, discrete vs. graded, can, in principle, be distinguished statistically, as they correspond to different classes of statistical models (Bartlema et al., 2014). The prediction of discrete individual differences corresponds to a latent mixture model, which assumes that the observed data are samples from a discrete set of probability distributions and estimates the parameters of each probability distribution and the proportion of data points that belong to each group. The prediction of graded individual differences corresponds to a mixed model, which assumes that every participant has their mean value, typically drawn from a Gaussian distribution of means. Finding that one of these models fits preferential-looking data better than the other would provide strong support for the Early Abstraction or Usage-Based accounts.

A related research strategy is to examine the source of individual variation in the IMPL task. Recall that Early Abstraction and Usage-Based accounts make different predictions about the relationship between the accumulation of lexical knowledge and grammatical competence. Usage-Based theories predict a pervasive relationship between the two, whereas Early Abstraction theories do not. In general, research on this question has tested the relationship between productive vocabulary and performance in the IMPL task. For example, Scott et al. (2018) found no relationship between productive vocabulary size and the comprehension of transitive sentences with novel verbs among 23-month-old children. On the other hand, Messenger and Fisher (2018) found that vocabulary size was related to the comprehension of 36-month-olds of passive sentences in the IMPL task. However, they argued that this finding was due to the relationship between vocabulary and lexical processing efficiency, the efficiency with which children recognize spoken words online (Fernald et al., 1998). Specifically, since children with larger vocabularies recognize words more efficiently than children with smaller vocabularies (Fernald et al., 2006), these children may identify words in the IMPL task more efficiently and, as a result, look at the target more reliably. Consistent with this argument, the authors found no relationship between productive vocabulary size and performance in the IMPL task in a follow-up study that minimized lexical processing efficiency demands.

However, the relevance of these findings in comparing Usage-Based and Early Abstraction accounts is unclear. First, it is not obvious that, from a Usage-Based account, total vocabulary is the most relevant measure of linguistic input. In particular, since the Usage-Based account assumes that children construct a transitive sentence schema by generalizing over a set of verb-specific constructions, the number of verbs a child knows should be a better predictor. While total vocabulary size should be correlated with the number of verbs known, the relationship is likely to be imperfect as children under 2 years exhibit a great deal of between-participant variability in the composition of their productive vocabularies (Mayor and Plunkett, 2014). Second, measures of vocabulary should be related to the acquisition of transitive sentences by children from an Early Abstraction account, as even from this perspective, children need to learn how abstract representations are marked and combined to create an inventory of constructions in their language. Therefore, vocabulary measures should predict which children have acquired construction and which have not, but should not account for variability within these groups. Any variability within the above-chance group should be due to non-syntactic factors and better accounted for by lexical processing efficiency than vocabulary. Any variability in the below-chance group should be completely random, as correctly identifying the target video presupposes relevant grammatical knowledge.

**Figure 1:**
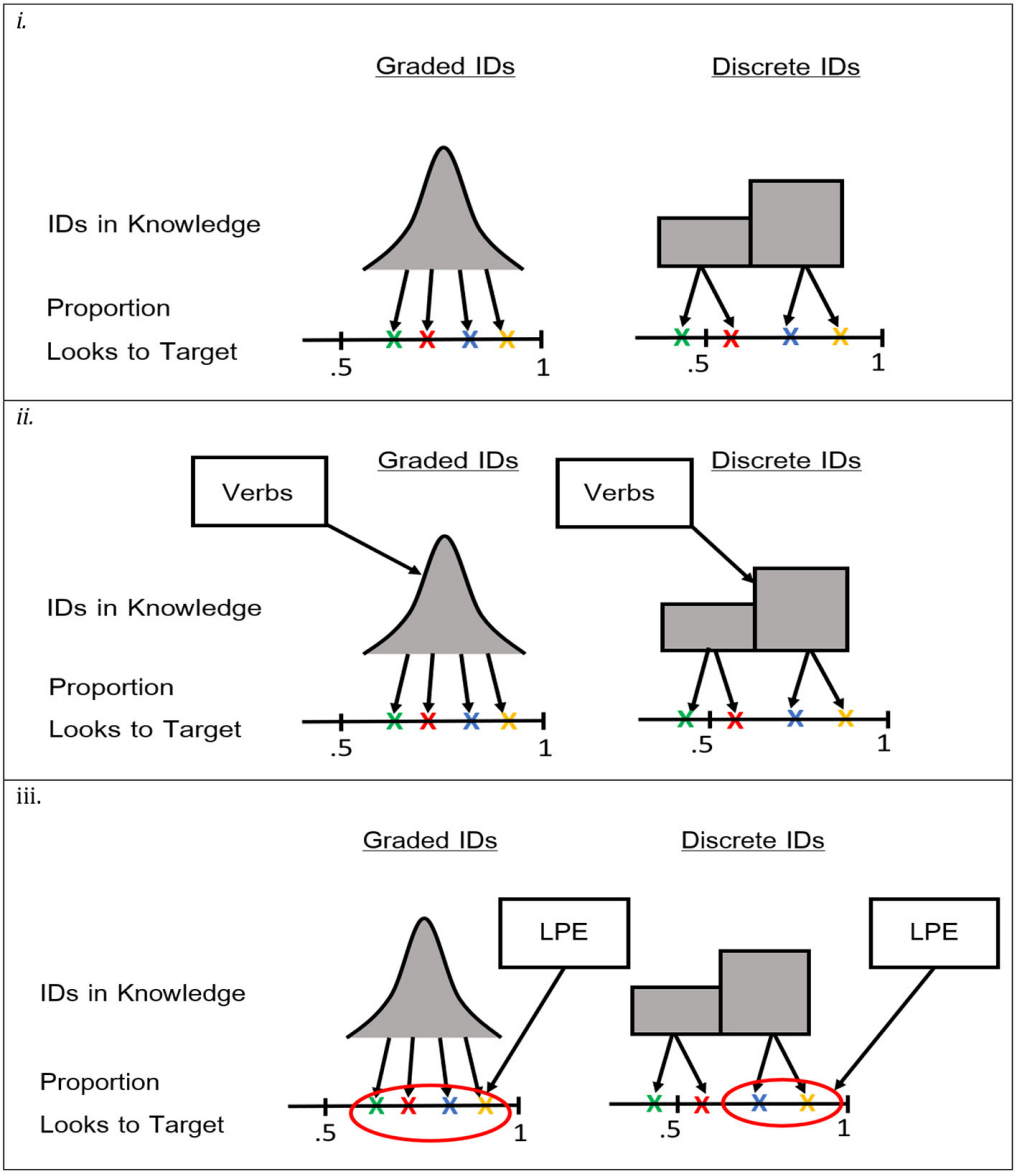
Depictions of the models testing predictions about the structure and source of individual differences. Xs represent hypothetical data points (proportion of looks to target) for different participants. Pane (i) represents models testing the structure of individual differences. Pane (ii) represents models testing the effects of the number of verbs known. Pane (iii) represents models testing the effect of lexical processing efficiency.

An alternative approach, then, is to use the number of verbs as the relevant input measure, including an explicit measure for lexical processing efficiency. Using the mixture and mixed models described above, the different predictions of Usage-Based and Early Abstraction accounts can be explicitly compared. In particular, to test the Usage-Based account, the number of verbs known and lexical processing efficiency can be added to the mixed model to see if they predict the mean proportion of looks to target. To test the Early Abstraction account, the mixture model can be used to test the predictions that the number of verbs known predicts the probability that a given child is in the above-chance group, and that lexical processing efficiency predicts variability within the above-chance group. These predictions are visualized in Figure 1.

This study reports on research aimed at addressing the questions above. In particular, we report on a large sample (*N* = 92) of 24-month-olds who completed a version of the IMPL task adapted from Gertner et al. (2006), as part of a large longitudinal study on language acquisition (Kidd et al., 2018b). It had three aims:

1) To replicate the finding that 24-month-olds look at the target video at above-chance levels and to determine whether these effects are apparent across both trials.

2) To examine the structure of individual differences in this task by comparing models that assume discrete and graded individual differences.

3) To examine the source of individual differences by adding the number of verbs known and a common measure of lexical processing efficiency to the models in (2).

## Method

### Participants

Participants came from the Canberra Longitudinal Child Language Project, a longitudinal study of language acquisition and processing from 9 to 60 months (Kidd et al., 2018b). Families were recruited from a medium-sized city in Australia. Inclusion criteria for the longitudinal study were: (i) full-term (at least 37 weeks gestation) babies born with a typical birth weight (>2.5 kg), (ii) a predominantly monolingual language environment, with the children acquiring Australian English as a first language [mean percentage of a language other than English = 2%, range: (0, 40%), mode = 0], and (iii) no history of medical conditions that would affect typical language development, such as repeated ear infections, visual or hearing impairments, or diagnosed developmental disabilities. Consistent with the demographics of the city, the sample was drawn from families of high socioeconomic status. Approximately 75% of the parents had completed a bachelor degree or higher. At 24 months, children completed an IMPL task based on that from Gertner et al. (2006), the looking-while-listening task (Fernald et al., 1998, 2006), and the MacArthur-Bates Communicative Inventory: Words and Sentences (Fenson et al., 2007). Of the 124 participants who completed at least one wave of testing, 115 completed the 24-month sessions. Four participants were later diagnosed with developmental difficulties and excluded, and 19 participants were excluded because of insufficient data in the IMPL task (See Results for more details). Therefore, 92 participants are included in the analyses below. They completed their 24-month testing session at a mean of 106.9 weeks of age (SD = 0.84 weeks, Min = 104.9 weeks, Max = 110.3 weeks). Of the 92 participants, 45 were female (49%).

### Materials

All the children completed the looking-while-listening (LWL) task prior to the IMPL task. The two tasks together took a combined 10 min (~6 min for the LWL task and ~4 min for the IMPL task) and were administered in a single session. Additionally, the parents of the participants completed the MacArthur Bates Communicative Development Inventory: Words and Sentences (Fenson et al., 2007) to measure vocabulary size.

#### IMPL Task

**Figure 2:**
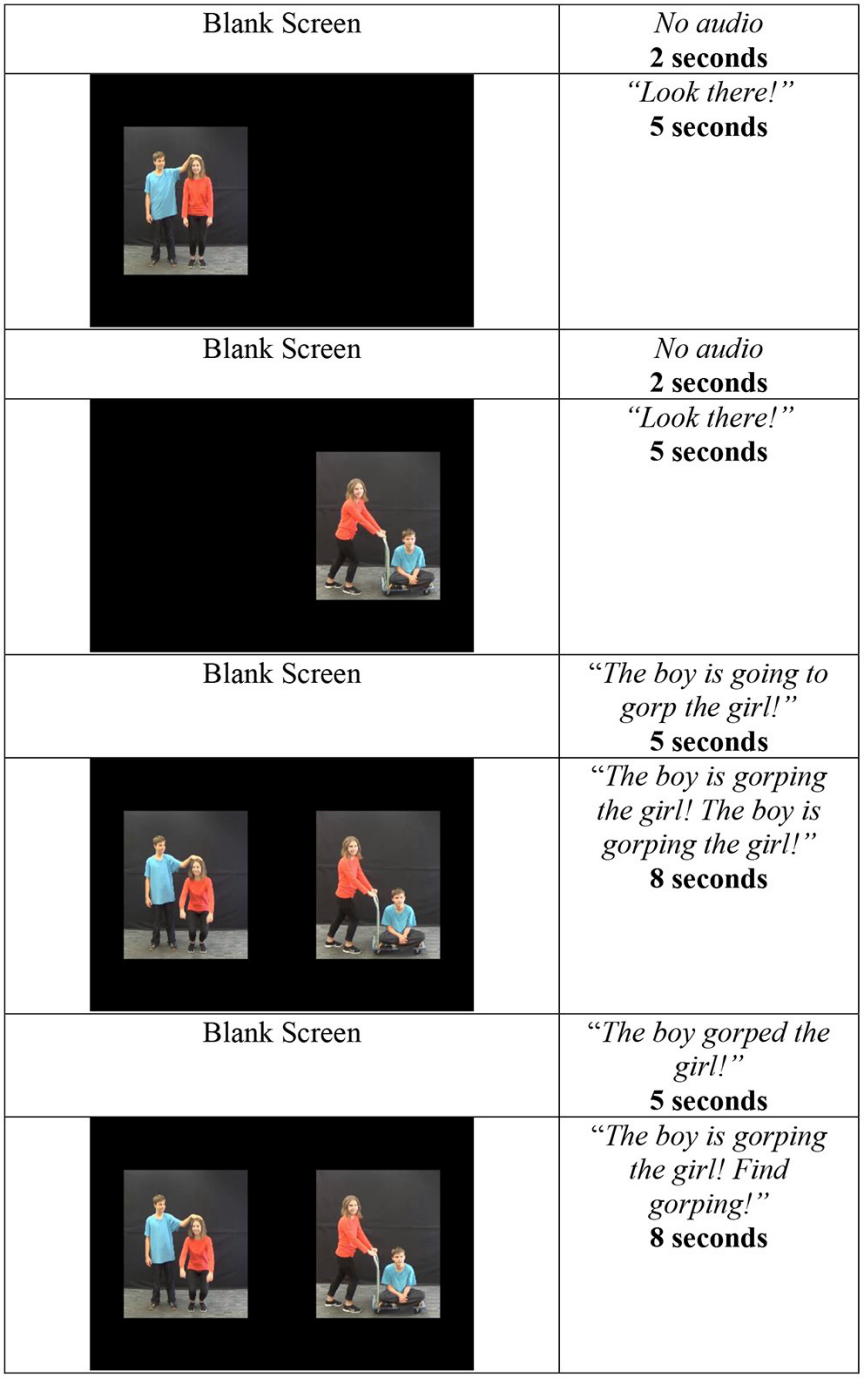
Structure of the Intermodal Preferential Looking (IMPL) task with novel verbs.

**Figure 3:**
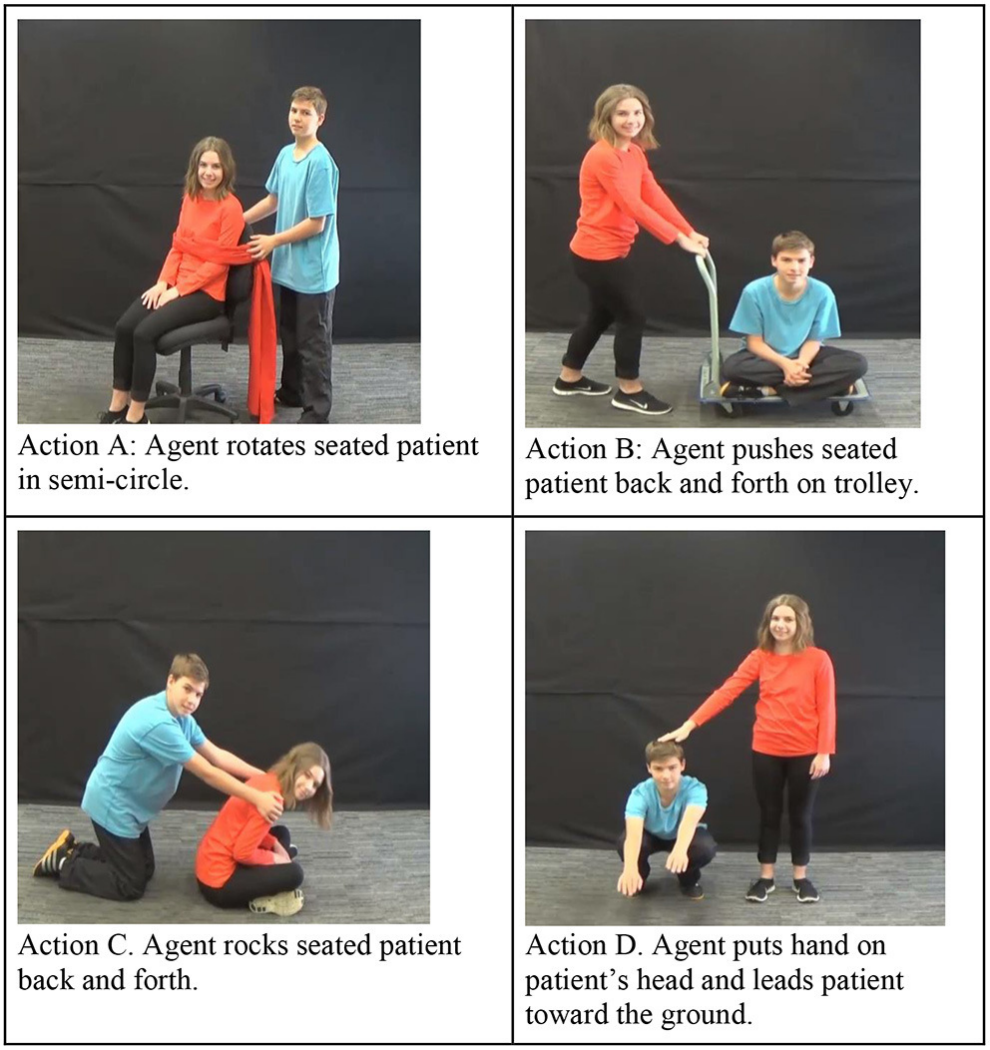
Actions used in the IMPL task.

The participants completed a version of the IMPL task described in Gertner et al. (2006), adapted for a Tobii T60XL (Tobii Pro, Stockholm, Sweden) eye-tracker, with sampling performed at a rate of 60 Hz. This task contained three phases: (i) character identification, (ii) familiar verb, and (iii) critical novel verb. The character identification and familiar verb phases served to prepare the participants for the critical novel verb phase. As such, we will describe the novel verb phase first. The novel verb phase is composed of two trials, and each is structured as depicted in Figure 2. At the beginning of each trial, children saw videos of two novel causal actions with opposite participant roles (see Figure 3 for all four actions). Each video played separately for 5 s. The participants then heard a transitive sentence with a novel verb (gorp or tam), and both videos played simultaneously. The videos played over two 8-s windows, across which the children heard the transitive construction with the novel verb a total of five times.

The participants saw a total of four novel causal actions, two as the target and two as the distracter, adapted from Gertner et al. (2006). In each of the two trials, one action served as the target (that is, its participant roles matched those conveyed in the sentence) and one action served as the distracter (that is, its participant roles mismatched those conveyed in the sentence). Several variables were balanced within participants, including the novel verb (gorp and tam), target side (right or left), target agent (the girl or the boy), and first video presented (target or distracter). We assigned the participants to one of the eight counterbalancing sequences (see Table 1 for details). Across participants, each action occurred equally often as the target and the distracter, and the agent approached the patient from the right side on equal numbers of trials. Across sequences, each target action occurred with two of the other actions as a distracter. All the video sequences are available on the Open Science Framework: https://osf.io/tqz8b/.

**Table 1:** Counterbalancing sequences for the Intermodal Preferential Looking (IMPL) task.

	**Trial 1**							**Trial 2**						
**Seq**	**Verb**	**Agent**	**Targ**	**Dist**	**Side**	**App**	**First**	**Verb**	**Agent**	**Targ**	**Dist**	**Side**	**App**	**First**
1	Gorp	Boy	D	C	Right	Left	Targ	Tam	Girl	A	B	Left	Left	Dis
2	Gorp	Boy	C	A	Left	Left	Dis	Tam	Girl	B	D	Right	Left	Targ
3	Gorp	Girl	B	A	Right	Left	Dis	Tam	Boy	C	D	Left	Left	Targ
4	Gorp	Girl	A	C	Left	Left	Targ	Tam	Boy	D	B	Right	Left	Dis
5	Tam	Girl	D	B	Right	Right	Targ	Gorp	Boy	A	C	Left	Right	Dis
6	Tam	Girl	C	D	Left	Right	Dis	Gorp	Boy	B	A	Right	Right	Targ
7	Tam	Boy	B	D	Right	Right	Dis	Gorp	Girl	C	A	Left	Right	Targ
8	Tam	Boy	A	B	Left	Right	Targ	Gorp	Girl	D	C	Righ	Right	Dis

*Each participant was assigned to one sequence*.

Prior to the test phase, the children completed a character identification phase and a familiarization phase. The character identification phase introduced the children to the two characters, the boy and the girl, who would be agents and patients in all of the subsequent actions. After the character identification phase, the participants completed a familiarization phase consisting of two trials with known verbs. These trials were created to familiarize the participants with the task and used videos of actions likely to be known by 24-month-olds (tickle, hug, wash, and feed). As these trials were not originally designed to test hypotheses, they were not as fully counterbalanced as the test trials. Actions were paired so that when *wash* appeared on one side of the screen, *tickle* appeared on the other, and when *hug* appeared on one side of the screen, *feed* appeared on the other. Additionally, the same actor served as the agent in both the target and distracter videos, and no actions were repeated within participants. To minimize possible training effects, we adapted the familiarization procedure from the no-training condition described in Dittmar et al. (2008a). The trials were structured similarly those shown in Figure 2, except that rather than relevant transitive sentences, the children heard “You are going to VERBing!,” “Where's VERBing? Find VERBing!,” and “You saw VERBing!” in the corresponding time windows. Attention-getter trials were included among all the trial types described above.

#### Looking-While-Listening Task

The looking-while-listening task was administered at 24 months (Fernald et al., 1998). These are the same data reported in the 24-month session of Donnelly and Kidd (2020). The participants saw images of 12 concrete objects (ball, bird, book, car, cat, dog, fish, shoe, apple, flower, frog, and teddy). On each trial, two images were presented on a 1,920-px × 1,200-px screen for 7,000 ms. The images were of approximately equal size and enclosed in 470-px × 450-px boxes at equal distances from the center of the screen. After ~2,000 ms, an audio file, recorded by a female native speaker of Australian English in child-friendly, natural speech, directed the children to the target image. The audio was timed so the target word began playing at 2,500 ms. The target word was introduced using one of three carrier phrases (“look at the,” “where is the,” and “find me the”). Across trials, each image occurred equally often as a target and a distracter, and they also occurred equally often on the left and right sides of the screen. To ensure that the responses were not due to the visual salience of one target (or distracter) image, across trials, two images were chosen for each word (again, each image occurred four times, two times as the target, and two times as the distracter). Four pseudo-randomized lists were created so that no target word was repeated within three trials and that the target image appeared on the same sidee of the screen in no more than two consecutive trials. Attention-getting fillers were played after every six trials. These were dynamic cartoons with encouraging audio (e.g., “Did you see it?!”) meant to keep the children engaged.

Lexical processing efficiency was measured using reaction times (RTs) by following the procedure in Fernald et al. (2006). Prior to calculating RTs, we removed trials in which the participants were looking at the screen for <50% of the 3,000-ms window between the onset of the target word and the offset of the image. Then, following Fernald and Marchman (2012), we calculated the duration to the first look at the target image for trials in which they were (a) looking at the distracter image prior to the target word and (b) shifted to the target image within 300 and 1,800 ms after the onset of the target word. The first look at the target image was defined as the first fixation of at least 100 ms to the target image.

For each child, the LWL task was conducted first, followed by the IMPL task, so that any order effects were common across the entire sample, as is common in individual differences studies.

#### The MacArthur Bates Communicative Development Inventory: Words and Sentences

The MacArthur Bates Communicative Development Inventory: Words and Sentences was administered at 24 months. This is a parental checklist of vocabulary knowledge that is widely used in the study of language acquisition (Fenson et al., 2007). The checklist was slightly modified to be appropriate for the Australian context (see Reilly et al., 2007) and contained 678 items.

### Analytic Strategy

All the models of preferential-looking data used the beta distribution as a likelihood function (Smithson and Verkuilen, 2006). The beta distribution is defined for continuous variables in the interval (0, 1), i.e., from 0 to 1 excluding exactly 0 and exactly 1. It is more appropriate than a normal distribution, as it accommodates the heteroskedasticity caused by floor and ceiling effects. For most of the analyses, raw proportions were used as the dependent variable. However, in analyses in which each observation was based on fewer eye-tracking samples (e.g., where looks within particular 2,000-ms time bins were the dependent variable), some proportions were equal to exactly 1 or exactly 0. In these cases, we applied the transformation described in Smithson and Verkuilen (2006)^3^. In all the models, we used the mean-precision parameterization of the beta distribution, which is characterized by a mean, μ, and a precision parameter, ϕ. The mean represents the central tendency of the distribution, and the precision represents the spread of the distribution at a particular central tendency. The variance of a beta distribution is the product of both of these two parameters, allowing for smaller variances near the ceiling and floor, while not forcing the variance to solely be a function of the mean (as in the binomial distribution). All the models were estimated in STAN (Stan Development Team, 2019), using the package brms (Bürkner, 2018).

## Results

All the analyses for this study, including additional analyses not reported, are available at https://rpubs.com/sdonnelly85/713289.

### Data Processing

One-hundred and eleven children completed the IMPL task and were not diagnosed with later developmental disabilities. Windows (four per child) with 66% missing data were excluded, and participants missing two or more windows were excluded, resulting in 92 participants. Additionally, one of eight sequences contained an incorrect audio file in one of the two 8-s windows for one of the trials. This sequence was corrected after seven participants had seen this sequence. This 8-s window was, therefore, removed for those seven participants.

### Descriptive Statistics

The mean proportion of looks to the target action in the IMPL task was only slightly above chance (*m* = 0.51, *SD* = 0.08). Of the 92 participants who completed the task, 50 looked to the target video more than 50% of the time. The mean RT on the LWL task from 24 months was 563 ms (*SD* = 111.7). The average productive vocabulary was 350.9 words (*SD* = 151.8), with an average of 54.6 verbs (*SD* = 30.2).

### Did Participants Look at the Target at Above-Chance Levels?

To examine whether participants looked to the target video at above-chance levels, we calculated the proportion of looks to the target video for each trial for each participant. We then analyzed these using a mixed-effects beta regression with random intercepts by the participant and by item (referring to each unique trial type in Table 1) using the default priors of brms. As beta regression uses a logit link function (wherein 0 corresponds to a probability of 0.5), the intercept in these models indicates how far the average participant differs from chance. The overall proportion of looks to the target did not differ from chance (logit scale: *b* = 0.02, *CI* = −0.12: 0.15, posterior probability = 0.61; probability scale: *Prop* = 0.5, *CI* = 0.47: 0.54).

**Figure 4:**
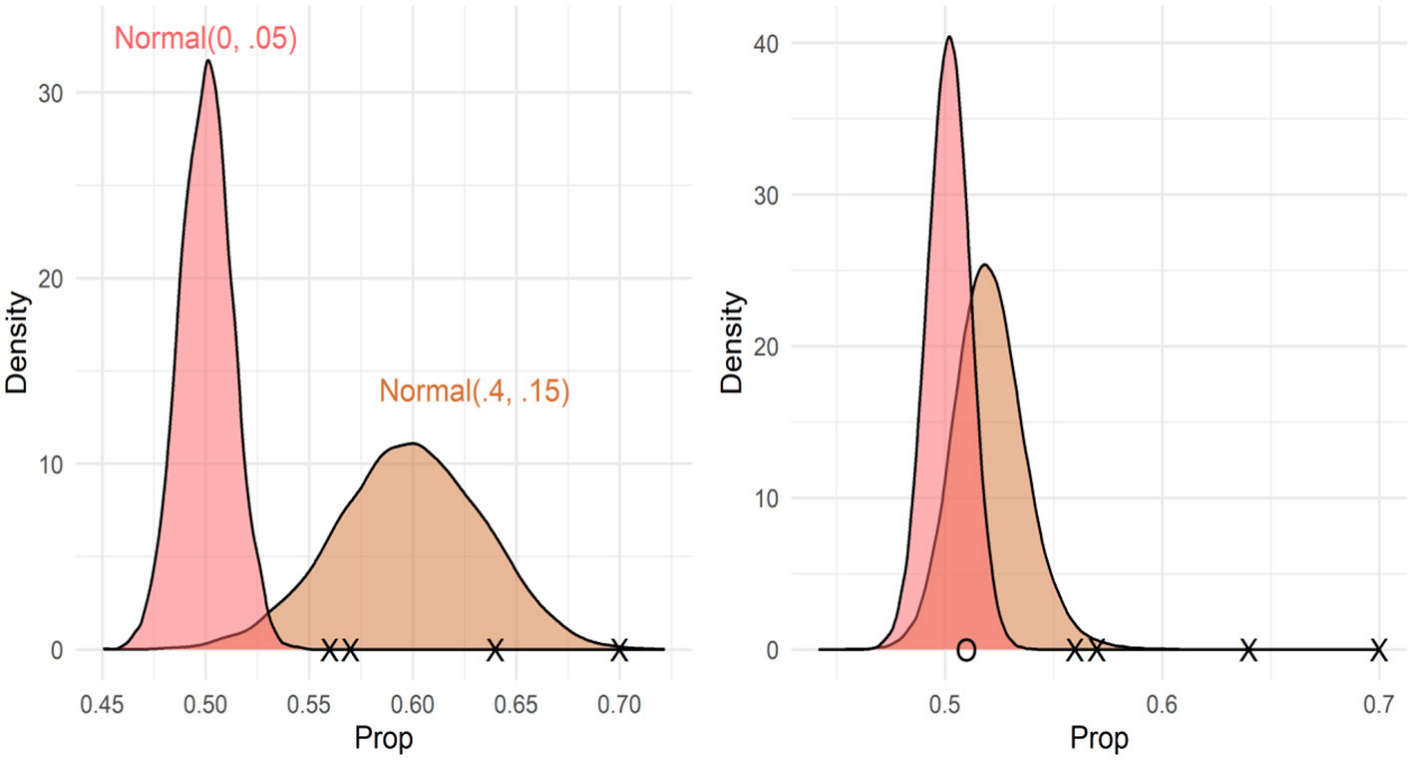
Prior (left pane) and posterior (right pane) distributions for Bayes factor analyses using Normal (0.4, 0.15) as the prior distribution. Xs represent the sample means from Gertner et al. (2006), and O (right pane only) represents the sample mean from this study. The Bayes factor comparing these models supported the null (BF = 0.04).

**Figure 5:**
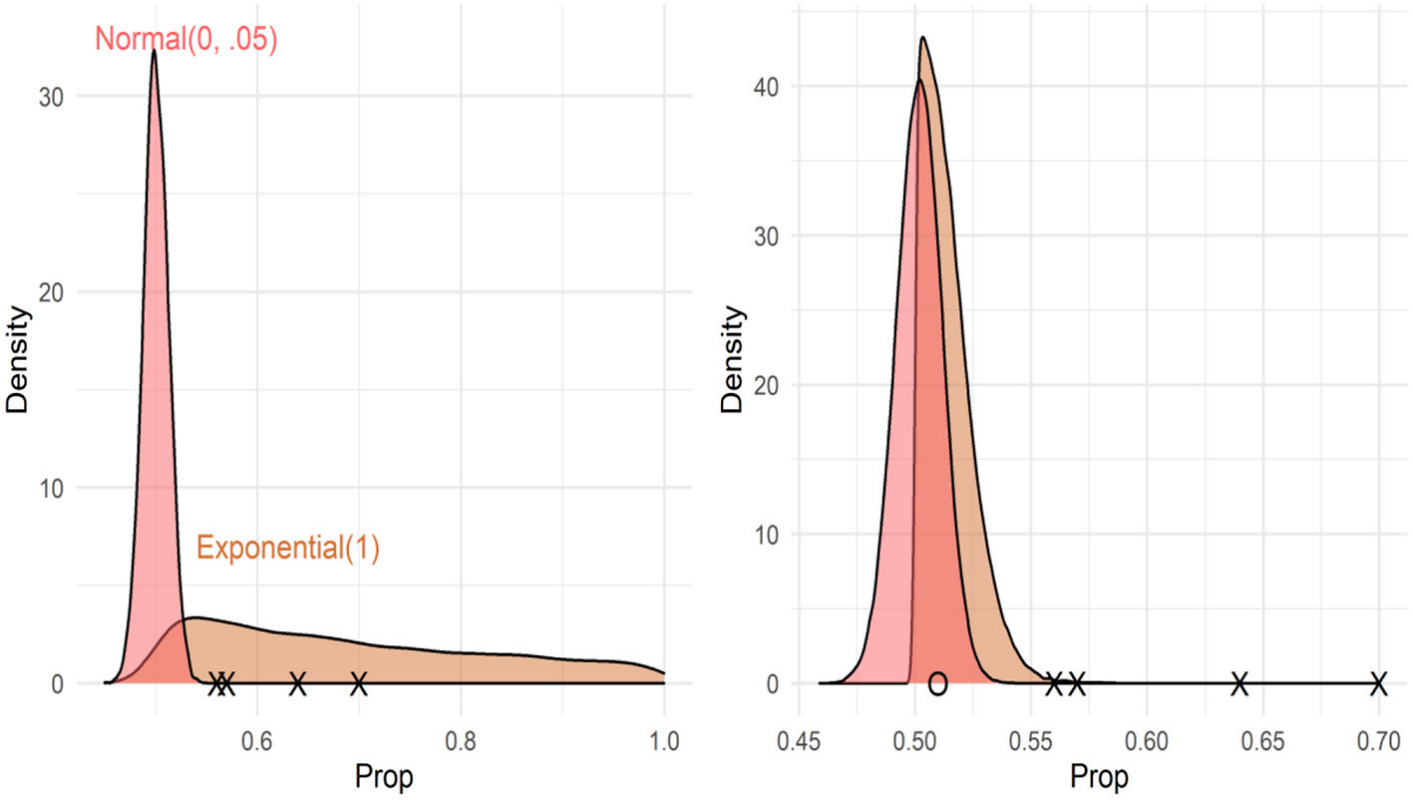
Prior (left pane) and posterior (right pane) distributions for Bayes factor analyses using Exponential (1) as the prior distribution. Xs represent sample means from Gertner et al. (2006), and O (right pane only) represents the sample mean from this study. The Bayes factor comparing these models supported the null (BF = 0.14).

Model coefficients and uncertainty estimates indicate the range of parameter values consistent with the data, but they cannot tell us whether the data are more consistent with a null or alternative hypothesis. To test whether the data were more consistent with a null (chance performance) or alternative hypothesis (above chance performance), we calculated the Bayes factors comparing null and alternative models. However, Bayes factors are strongly influenced by the choice of priors, and care must be taken to choose priors consistent with each hypothesis. For the null hypothesis, we used a normal distribution with a mean of 0 and an SD of 0.05. This assumes that there is a 95% chance that the true proportion of looks to the target video is between 0.48 and 0.52. We considered two different alternative hypotheses, a normal distribution with a mean of 0.4 and a standard deviation of 0.15, which assumes that there is a 95% chance that the true proportion of looks to the target is between 0.54 and 0.66. This distribution was chosen to be consistent with the sample means from Gertner et al. (2006). See Figure 4, left pane, which compares the null and alternative priors for this comparison. We also considered an exponential distribution with a rate parameter of 1, which assigns near-uniform probabilities to proportions between 0.5 and 1 (assuming a 95% chance that the true mean falls between 0.51 and 0.95). See Figure 5. We calculated the Bayes factors comparing each of these alternative hypotheses to the null hypothesis using bridge sampling (Gronau et al., 2017). This revealed that the data were ~30 times more likely under the null hypothesis than alternative hypothesis 1 (BF = 0.034) and ~8 times more likely under the null hypothesis than alternative hypothesis 2 (BF = 0.121). In sum, the proportion of looks to the target video, when summed across time windows, was more consistent with the hypothesis that participants were not looking at above-chance levels.

An examination of the box plots revealed that Action C attracted slightly more looks than the other actions when it was both the target and the distracter. We ran two additional sets of analyses to see if this could explain the pattern of results above. First, we estimated the proportion of looks to the target action when we excluded trials in which Action C was the target or distracter (*BF* = 0.08 and 0.17, respectively). Second, we explicitly controlled for target salience, as follows. Recall that each action was presented once to each participant as either the target or the distracter. We calculated the proportion of looks each action attracted when it was the distracter video. We then logit-transformed this proportion and included it in a model testing the overall looks of participants to the correct action. Including this effect allowed us to interpret the intercept as the increase in looks to the target action when it was the target relative to when it was a distracter. The new intercept did not differ from 0 (*b* = 0.05, *CI* = −0.08: 0.18, posterior probability = 0.80). It was not possible to estimate Bayes factors in this context because it was not clear what the priors should be for the intercept in this context.

### Do Participants Look Above-Chance Within Particular Windows?

**Figure 6:**
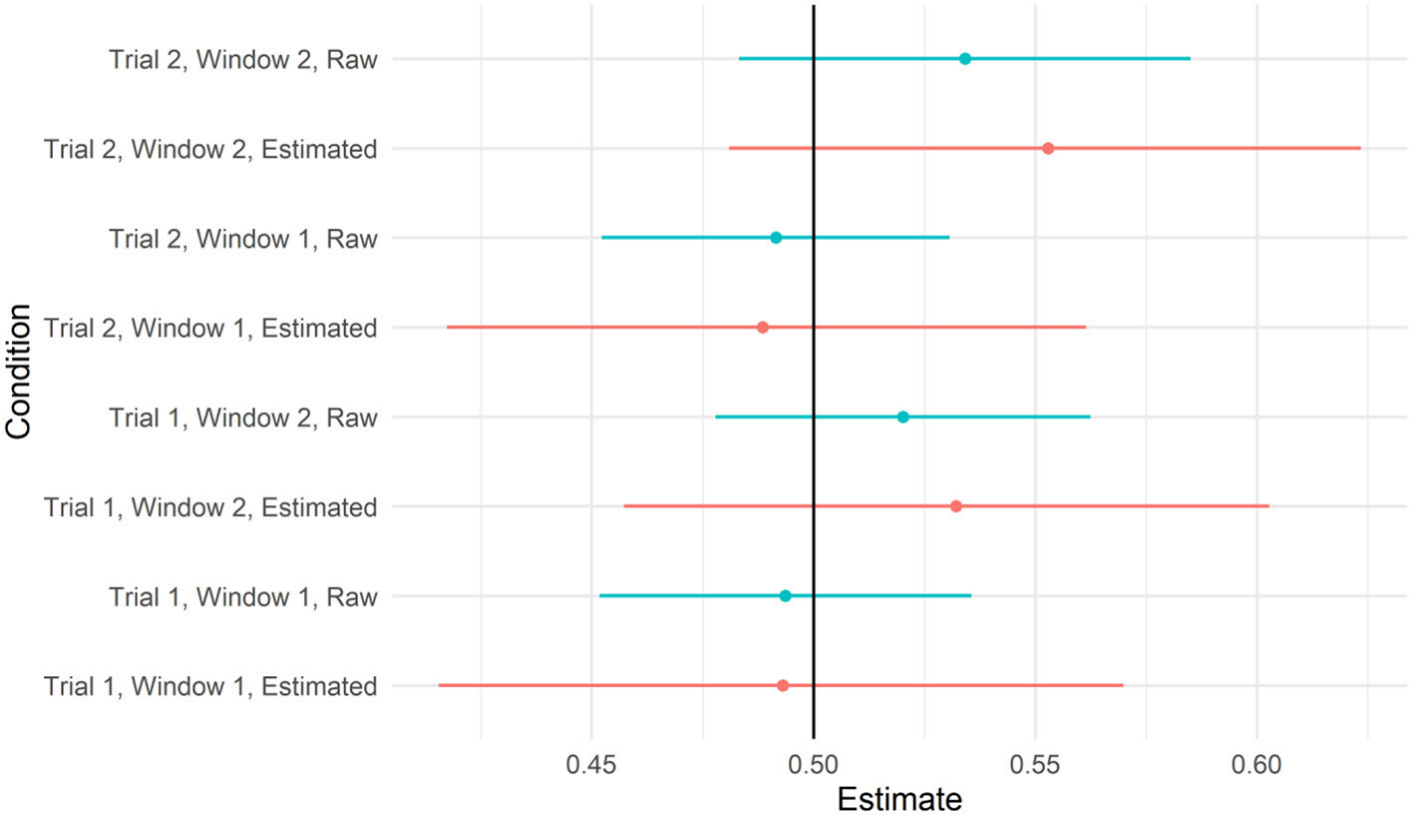
Predicted values from the model testing interaction between trial and window and 95% credible intervals. Raw means and 95% CIs are plotted alongside for reference.

We next asked whether there was evidence that participants looked to the target video at above-chance levels in some time windows. Recall that the IMPL task included two trials, one for each novel verb, and each trial contained two 8-s time windows. We, therefore, calculated the proportion of looks to the target within each 8,000-ms window within each trial, and fit a mixed-effects beta regression with fixed effects for target salience (described above), window and trial (both sum coded, with Window 1 and Trial 1 both set to 0.5), and the interaction between window and trial to these data (assuming full, uncorrelated random effects by participant and item). To determine whether the proportion of looks to the target differed from chance in any of these conditions, we plotted the model-predicted means and credible intervals from each condition in Figure 6, with empirical means and confidence intervals, calculated on the raw data for each window separately, for reference. As can be seen, the credible intervals for all four conditions overlapped with 0.5. For the full set of parameter estimates, see the accompanying html file.

**Figure 7:**
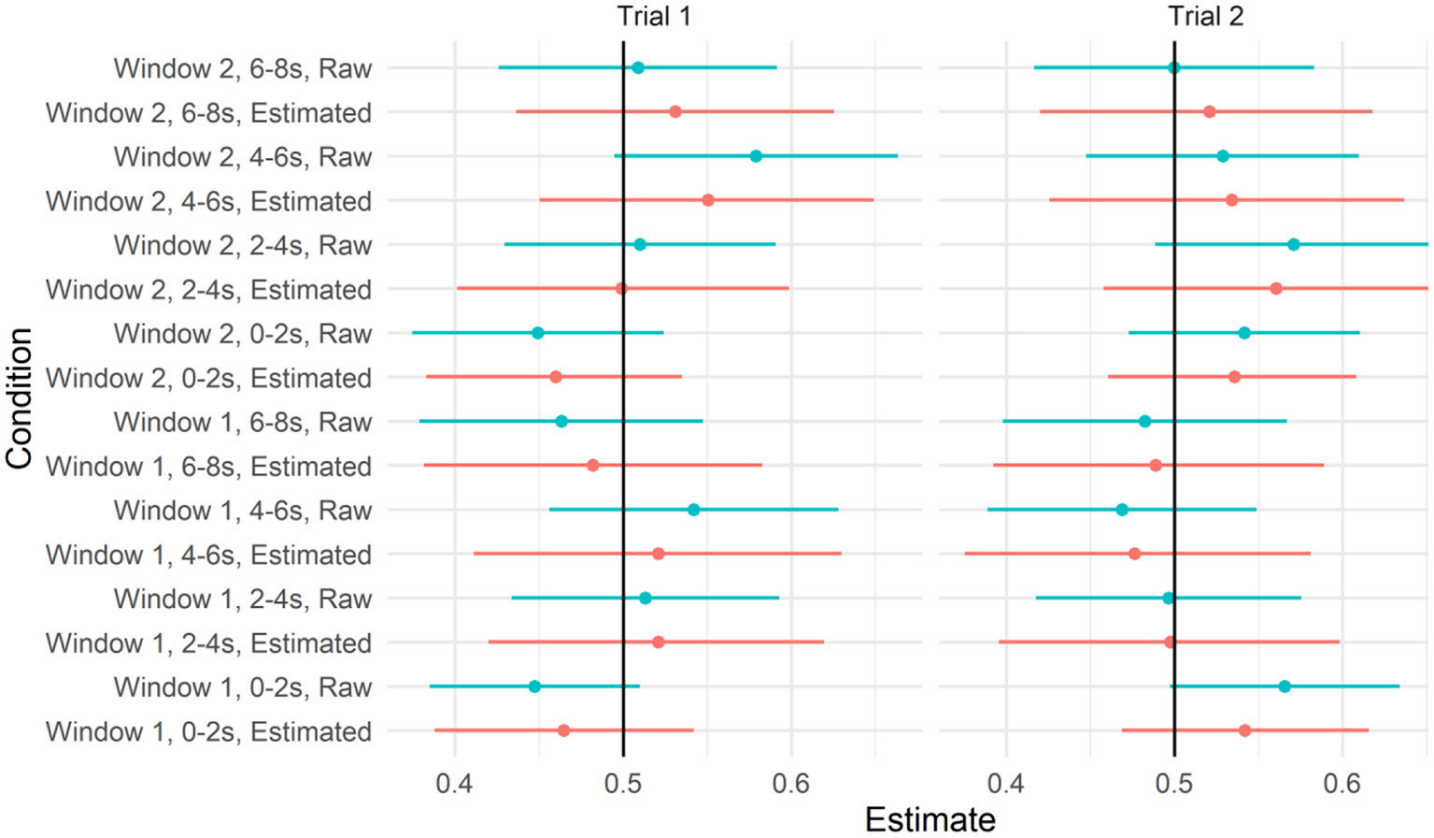
Predicted values from the model testing the interaction between trial, window, and time bin and 95% credible intervals. Raw means and 95% CIs are plotted alongside for reference.

To recreate the sort of analyses reported by Dittmar et al. (2008a), we further disaggregated the data by 2,000-ms time bins, within the time window and trial. We calculated the proportion of looks to the target video within each of these 2,000-ms bins and applied the transformation from Smithson and Verkuilen (2006) to remove 0 s and 1 s. We fit a model with a three-way interaction between window, trial, and bin, with full uncorrelated random effects by the participant and by item. We plotted the model implied means and credible intervals for each of these conditions, along with the raw data in Figure 7. As can be seen, the model did not predict the above-chance looks to the target video in any of the time bins. For the full set of parameter estimates, refer to the accompanying R Markdown file.

**Figure 8:**
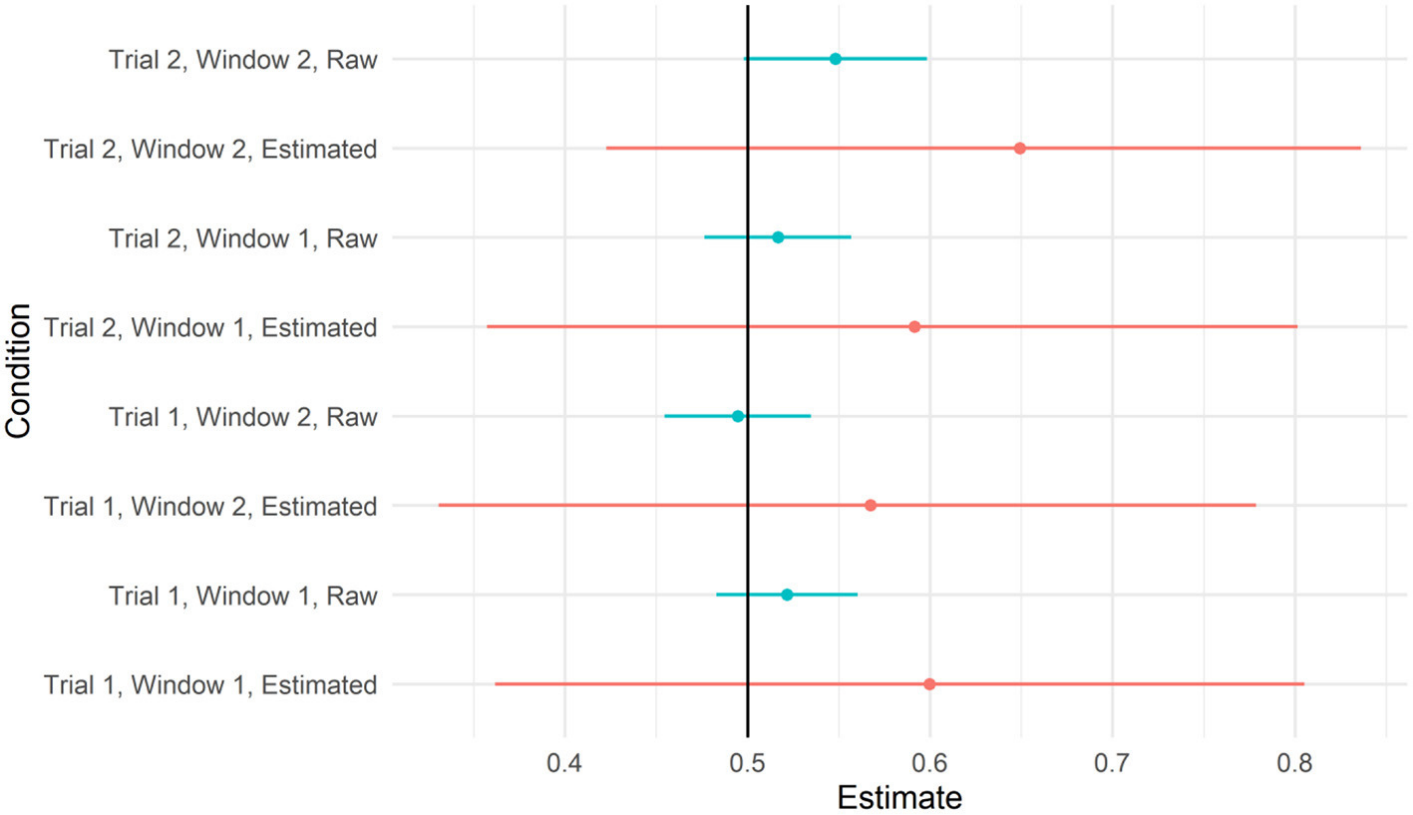
Predicted values from the model testing the interaction between trial and window 95% credible intervals when action C was removed. Raw means and 95% CIs are plotted alongside for reference.

**Figure 9:**
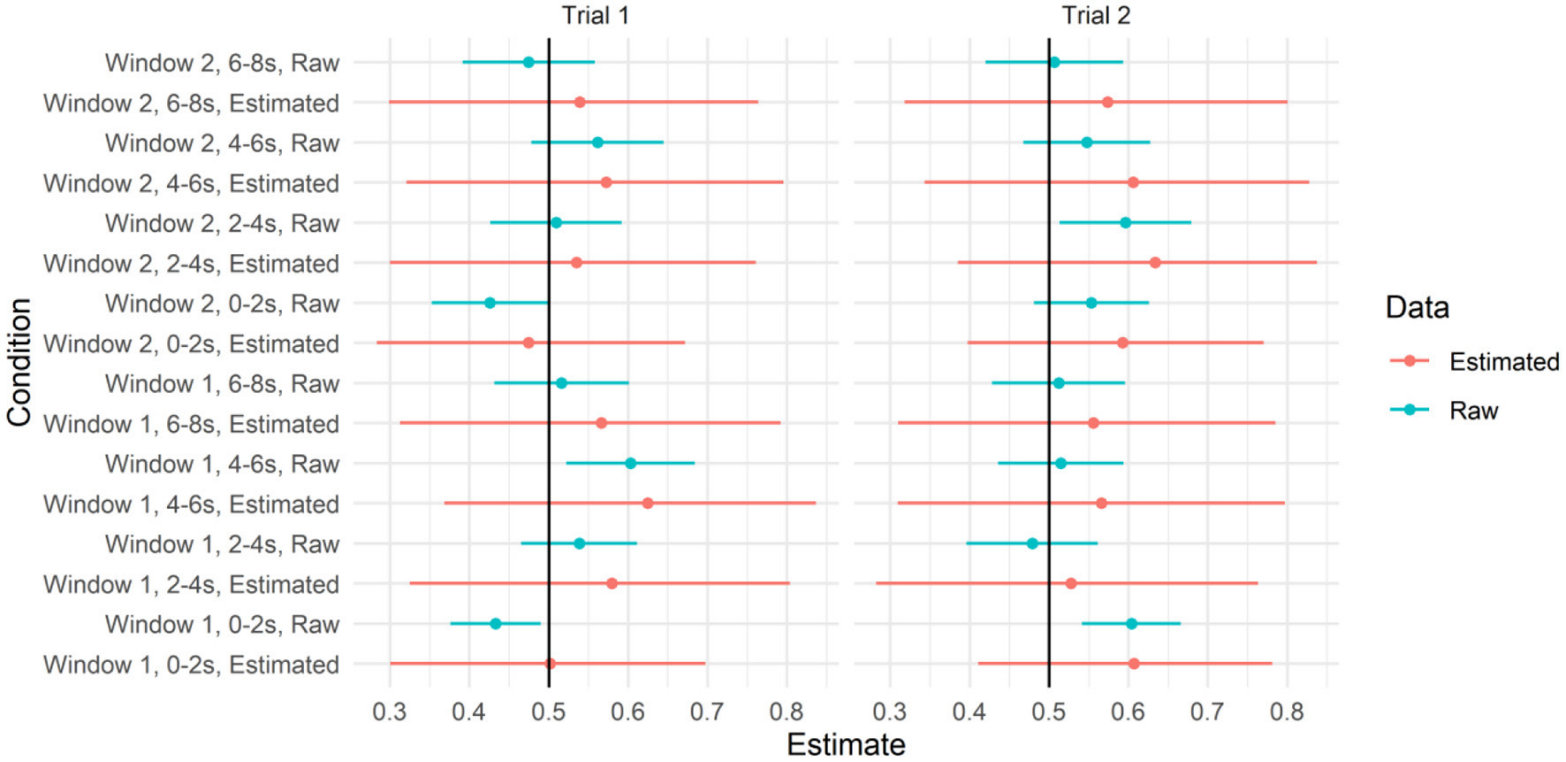
Predicted values from the model testing interaction between trial, window, and time bin and 95% credible intervals when action C was removed. Raw means and 95% CIs are plotted alongside for reference.

As with our previous analyses, we re-ran these models without Action C. We re-created the plots above on this reduced data set (refer to Figures 8, 9). As can be seen, the estimates of the model of looks to the target did not differ from chance in any of these time windows. Unlike in Figure 7, the raw means for some time windows had confidence intervals that did differ from chance. However, given that (a) these estimates were based on a subset of data and (b) their errors do not account for the dependence between observations, we conservatively view these effects as false positives.

### Graded vs. Discrete Individual Differences

To estimate the models of discrete and graded individual differences, we fit mixture and mixed models to the IMPL data. We used the average proportion of looks to the target across all time windows, rather than including multiple observations per participant, as the dependent variable. We did this because the correlations between observations within participants were surprisingly low: The correlation between trials within participants was negative and significant (*r* = −0.219, *p* < 0.05). The correlation between windows (collapsing across trials) was non-significant (*r* = −0.05, *p* > 0.05), though this likely reflects the fact that participants switch between target and distractor throughout trials and, at this age, may do so in idiosyncratic ways. When averaged within windows and trials, all correlations were non-significant (*r*s ranged between −0.17 and 0.07). Including multiple observations per participant when correlations were this low would have likely been problematic for the model of discrete individual differences. Such a model would include at least two unobserved variables: the probability that a given participant belongs to the above-chance group and the variance of participant means within each of those groups. Given these correlations, the latter parameters would be difficult to identify, and even if they were identified, these models would be extremely difficult to interpret. We used this data point as the dependent variable for the model of graded individual differences as well, to make these two models comparable.

The model of graded individual differences was an intercept-only model with Gaussian random effects by participant, as in the equations:

Prop_i_~ Beta(μ_i_, ϕ)

μ_i_ = inverse.logit(B_0_ + t_i_)

t_i_~ Normal(0, σ)

**Figure 10:**
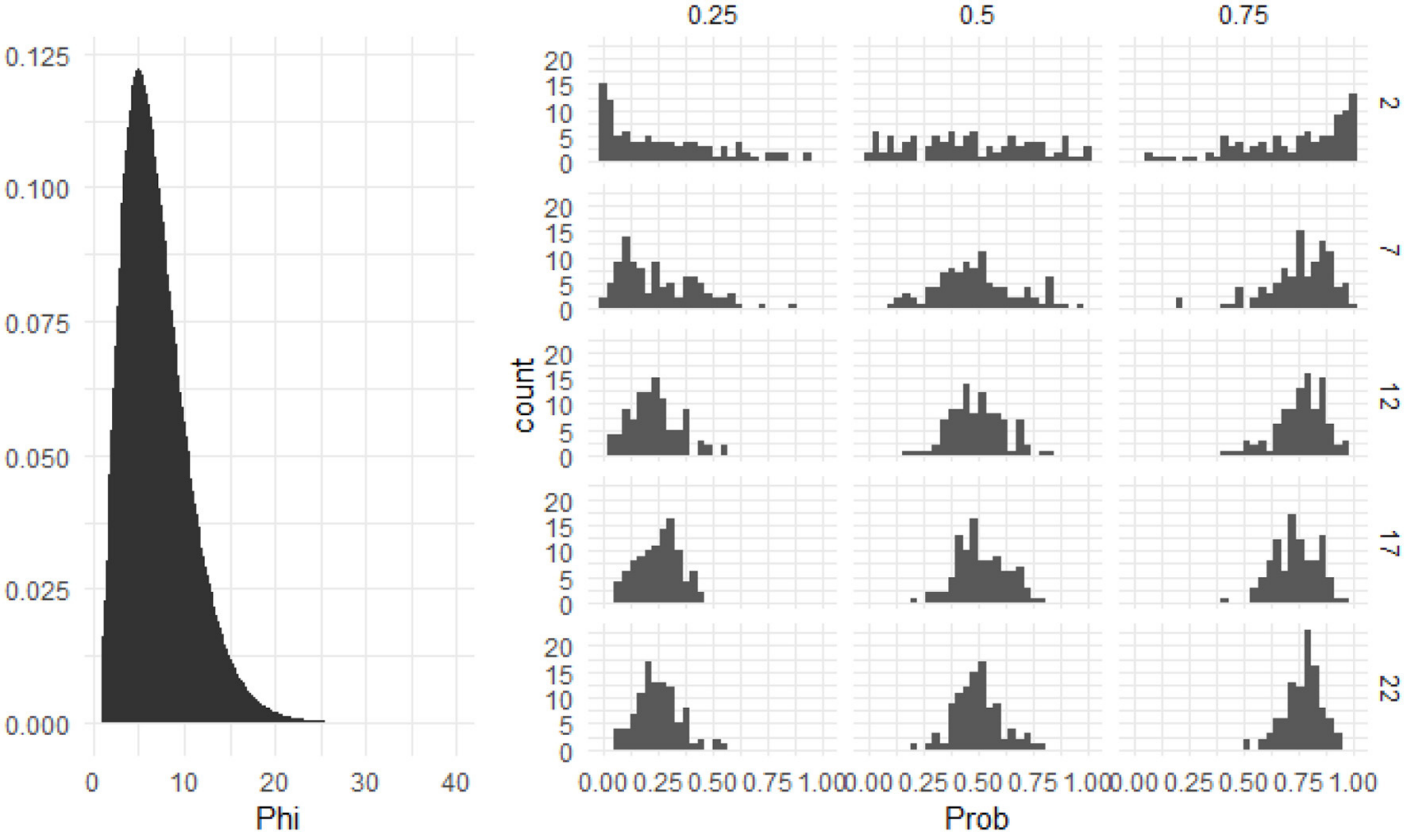
The prior distribution of the precision parameter for models of individual differences. The left side shows the prior density on the precision parameter, phi. The variance of a beta distribution is a product of its mean and phi. The right side shows the histograms of simulated data assuming varying means and precisions (the latter of which span the range of plausible values encoded by the prior distribution). Columns represent different values of the mean, and rows represent different phis. As can be seen, this prior is consistent with data of varying spreads.

This model contains two variability parameters: a random intercept variance, which represents variability among participant-level means, and a precision parameter, which represents how variable data points are around their predicted means. Because we had to limit our analysis to one data point per participant, it was important to choose informative priors for these parameters. For σ, the random intercept distribution, we chose Normal(0, 1). For ϕ, we chose Gamma(3.5,0.5), as it provided a satisfactory coverage of plausible values of ϕ in the IMPL data. Figure 10 shows the prior density of this parameter on the left and nine histograms of randomly generated data assuming values of ϕ across the range of plausible values implied by the prior distribution. As can be seen, this prior distribution can flexibly accommodate the type of data one would expect to see in an IMPL task. The parameter estimates and 95% credible intervals for this model are in Table 2. As can be seen, overall looks did not differ from 0, and the between-subject variance was quite small.

**Table 2:** Parameter estimates for models of graded individual differences.

**Parameters**	**Structure**	**Source: LWL RT**	**Source: Verbs**
Intercept	0.04 (−0.05: 0.13)	0.03 (−0.06: 0.12)	0.14 (−0.04: 0.33)
Random effect	0.08 (0.00: 0.22)	0.08 (0.00: 0.22)	0.08 (0.00: 0.22)
LWL RT		0.06 (−0.03: 0.15)	
Verbs			−0.17 (−0.48: 0.13)
Phi	22.70 (16.61: 30.32)	22.51 (16.32: 30.20)	22.52 (16.37: 30.28)

*95% credible intervals in parentheses. All statistics are on the logit scale*.

The model of discrete individual differences was a mixture model of beta distributions. This model took the form:

Prop_i_~ (1-T_i_)^*^Beta(μ_1_, ϕ_1_) + ${\text{T}}_{\text{i}}^{*}$Beta(μ_2_, ϕ_2_).

T_i_~ Bernoulli(π)

This model assumes that every observation belongs to one of two groups, with different mean and precision parameters, and simultaneously models those parameters and the proportion of observations belonging to each of the two groups. We assumed the same prior on ϕ_1_ and ϕ_2_ as we did on ϕ in the model of graded individual differences, and we assumed the same priors on μ_1_ and μ_2_ as we did when calculating Bayes factors comparing the null and alternative hypotheses. Parameter estimates and 95% credible intervals from this model can be seen in Table 3. As can be seen, the model detected two groups, one looking at chance (*m* = 0.5, *CI* = 0.48: 0.52, converted to a probability scale) and one looking at above chance (*m* = 0.58, *CI* = 0.55: 0.65, converted to a probability scale). However, the estimate of the proportion of participants in the above-chance group was low with high uncertainty (*Prop* = 0.19, *CI* = 0.02: 0.85).

**Table 3:** Parameter estimates for models of discrete individual differences.

**Parameters**	**Structure**	**Source: LWL RT**	**Source: Verbs**
Looks to Target Group 1	0.01 (−0.07: 08)	0.00 (−0.07: 0.07)	0.02 (−0.06: 0.09)
Looks to Target Group 2	0.33 (0.15: 0.62)	0.34 (0.07: 0.64)	0.36 (0.07: 0.66)
Proportion of Sample inGroup 2	0.17 (0.02: 0.81)	0.13 (0.01: 0.45)	−2.70 (−6.18: 2.68)
Phi Group 1	23.31 (12.32: 32.14)	23.39 (15.86: 32.17)	22.16 (8.89: 30.90)
Phi Group 2	11.41 (3.50: 24.21)	10.70 (3.26: 22.18)	9.93 (2.59: 23.34)
LWL RT		0.13 (−0.59: 0.69)	
Verbs			−0.06 (−1.05: 0.91)

*95% credible intervals in parentheses. All statistics are on the logit scale*.

While it is possible to compare these models using Bayes factors, they are greatly influenced by the choice of prior. Since we chose relatively strong priors to make the models estimable, we compared the models by leave-one-out cross-validation, a method for comparing the predictive accuracy of two models (Vehtari et al., 2017). This statistic measures the out-of-sample predictive accuracy of the two models. This suggested a numerical preference for the model of graded individual differences (*diff* = −0.2); however, this value was smaller than its standard error (*SE* = 0.6), suggesting that the difference was not reliable.

### Sources of Individual Differences: Lexical Processing Efficiency

To test the predictions the two accounts make about the relationship between lexical processing efficiency and the proportion of looks to the target, we augmented the two above models in the following way^4^. We added a regression coefficient for the LWL RT to the model of graded individual differences in the following manner:

Prop_i_~ Beta(μ_i_, ϕ)

μ_i_ = inverse.logit(B_0_ + ${\text{B}}_{1}^{*}$LWL_RT_i_ + t_i_)

t_i_~ Normal(0, σ)

For the model of discrete individual differences, we added a regression coefficient LWL RT to the mean of the second group, as follows,

Prop_i_~ (1-T_i_)^*^Beta(μ_1_, ϕ_1_) + ${\text{T}}_{\text{i}}^{*}$Beta(μ_2i_, ϕ_2_),

μ_2i_ = inverse.logit(B_0_ + ${\text{B}}_{1}^{*}$LWL_RT_i_)

T_i_~ Bernoulli(π)

Parameter estimates from these models are presented in Tables 2, 3, respectively. As can be seen, lexical processing efficiency was not related to the proportion of looks to the target in the manner predicted by either account.

### Sources of Individual Differences: Knowledge of Verbs

To test the predictions the two accounts make about the relationship between the number of verbs known and the proportion of looks to the target, we augmented the mixed and mixture models in the following way^5^. We added a regression coefficient for the number of verbs known to the model of graded individual differences in the following manner:

Prop_i_~ Beta(μ_i_, ϕ)

μ_i_ = inverse.logit(B_0_ + ${\text{B}}_{1}^{*}$Verbs_i_ + t_i_)

t_i_~ Normal(0, σ).

For the model of discrete individual differences, we added a regression coefficient for verbs known to the probability that a given participant belonged to the second group in the following manner:

Prop_i_~ (1-T_i_)^*^Beta(μ_1_, ϕ_1_) + ${\text{T}}_{\text{i}}^{*}$Beta(μ_2_, ϕ_2_),

T_i_~ Bernoulli(π_i_),

π_i_ = inverse.logit(B_0_ + ${\text{B}}_{1}^{*}$ Verbs_i_).

The parameter estimates from these models are presented in Tables 2, 3, respectively. As can be seen, the number of verbs known was not related to the proportion of looks to the target in the manner predicted by either account.

### Are the Familiar-Verb Trials Subject to Individual Differences?

Given the ambiguous results of our test trials, we conducted a set of exploratory analyses on our practice trials to determine whether our task was, in principle, capable of detecting individual differences. For our instantiation of the IMPL Task, to be a measure of individual differences in our sample, we would expect three conditions to hold. First, performance in the task should reflect competence in the relevant domain. Second, performance across trials, which presumably measure the same construct, should be correlated. Third, performance in the trials should be related to theoretically relevant predictor variables. All of these analyses with their results are available online (https://rpubs.com/sdonnelly85/780313). All procedures in this section are analogous to those in the prior section unless stated otherwise.

To test the first condition, we tested whether the overall looks to the target action differed from chance. Looks to the target were above chance, although the credible interval greatly overlapped with 0 (logit scale: b = 0.14, CI = −0.15: 0.44; probability scale: Prop = 0.53, CI = 0.47: 0.61, posterior probability = 0.84). Using the same priors as the main results, we calculated Bayes factors comparing null with alternative hypotheses and found a numerical, although trivial, preference for the null to both alternative hypotheses (BF = 0.46 and 0.42, respectively). As was the case with the test trials, the participants looked to some actions more than others, whether they occurred as targets or distracters. To control for these preferences, we calculated the proportion of looks to the relevant action when it occurred as the distracter and logit-transformed this value. When this variable was added to the model, the overall proportion of looks to the target action was positive, with a credible interval that did not overlap with 0 (logit scale: b = 0.17 CI = 0.03: 0.3, posterior probability = 0.99; probability scale: Prop = 0.54, CI = 0.51: 0.57). Thus, there was evidence that the participants looked to the target action at above-chance levels, although only when action preference was controlled for. However, we note that this effect is smaller than what is typically reported in IMPL studies.

To address the second condition, we calculated the correlations between trials and windows, as we did in the Results section. When the data were disaggregated by the window, there was a moderately significant correlation between windows 1 and 2 (*r* = 0.383, *p* < 0.001). When the data were disaggregated by trial, the correlation between trials 1 and 2 did not significantly differ from 0 (*r* = 0.027, *p* > 0.05). When the data were disaggregated by window and trial, there were significant correlations between windows 1 and 2 in both trials 1 and 2 (*r* = 0.417, *p* < 0.001 and *r* = 0.372, *p* < 0.001, respectively), but not between trials 1 and 2 within either window (*r* = −0.082, *p* < 0.05 and *r* = 0.014, *p* < 0.05, respectively).

To address the third condition, we considered whether performance in the practice trials was related to either of the predictor variables, LWL RT, or the proportion of verbs known. To test this, we fitted two additional models, augmenting the one that controlled for target preference described above, by adding LWL RT and the proportion of verbs known, respectively. The effect of LWL RT was negative, although its credible interval greatly overlapped with 0 (*b* = −0.01, *CI* = −13: 0.12, posterior probability = 0.54). The effect of the proportions of verbs known was positive, with a credible interval that did not overlap with 0 (*b* = 0.42, *CI* = 0.06: 0.81, posterior probability = 0.99). Thus, the children who knew more verbs looked to the target video at above-chance levels than those who knew fewer.

In sum, there was moderate evidence for all three conditions. First, there was some evidence that children looked at the target video at above-chance levels, although the magnitude of this effect was small and dependent upon controlling for the target preference. Second, the probabilities of looks to the target were correlated across consecutive 8-s time windows, but not across trials. Third, performance in the task was positively related to the size of verb vocabularies of the children.

## Discussion

This study aimed to determine whether: (a) participants could correctly interpret transitive sentences with novel verbs, (b) this effect was restricted to certain time windows, (c) participants exhibited discrete or graded individual differences in their ability to comprehend transitive sentences with novel verbs, and (d) individual differences were predicted by the number of verbs children knew and/or their lexical processing efficiency. We found that, overall, the participants did not look to the target at above-chance levels, and found very little evidence that they did so within specific time windows. Moreover, our data did not provide strong evidence in favor of either of the two models of the structure of individual differences and did not support any of the predictions about the source of individual differences. Given the ambiguous pattern of the results, we examined performance in our practice trials to determine whether our task was, in principle, capable of capturing meaningful individual differences, and found moderate evidence for this proposition. We discuss these results and their implications for using the IMPL data in the current modeling framework below.

We found moderate evidence against the hypothesis that, on average, 24-month-olds can comprehend a novel verb in a transitive sentence structure. This null pattern in the results remained even when we controlled for target preference in two ways: by excluding an action that attracted a disproportionate number of looks as the target and the distracter and by including the proportion of looks to the relevant action when it was the distracter as a control variable. Our results are inconsistent with those of Gertner et al. (2006), who found that samples of 21- and 25-month-olds looked to the target at above-chance levels, but are consistent with the no-training condition of Dittmar et al. (2008a), in which a sample of 21-month-olds did not look to the target at above-chance levels without the help of familiarization trials. Given that we conducted training trials similar to those in Dittmar et al. (2008a), our results seem to reinforce their conclusions that the success of toddlers in comprehending transitive sentences with novel verbs is contingent on their immediately preceding linguistic experience. However, our findings also appear to contradict those of Scott et al. (2018), who, in one of their two studies, did not include any familiarization trials with 23-month-olds. It is difficult to identify the exact cause of the discrepancy between these studies, as they differed on multiple dimensions; in particular, Scott et al. used animated videos with non-agent subjects and non-causative actions.

Moreover, we did not find evidence that the participants looked to the target at above-chance levels in some of the windows. When we calculated proportions within each 8-s window, the model did not find evidence that the participants looked to target action at above-chance levels within any window. When raw data were compared to chance (and one action was removed), there was some evidence that the participants looked at above-chance levels in three 2-s windows. However, given that those confidence intervals do not fully account for the uncertainty in the data, there is a distinct possibility that these results are false positives.

Our results align with the observation of Ambridge and Lieven (2015) that performance in the IMPL task with novel verbs is quite variable across different instantiations of the task. Such a conclusion is consistent with Usage-Based theories. For example, Abbot-Smith and Tomasello (2006) and Ambridge and Lieven (2015) note that we would expect such variability if children have fragile, tentative representations of a syntactic structure that are suitable for some tasks but not others. However, these findings could also be explained by Early Abstraction theories, if one assumes that non-syntactic processing demands are sufficiently variable across instantiations of the task. It is worth noting, then, that our study differed from previous ones by including an additional eye-tracking task (The LWL task). The inclusion of the LWL task raises the possibility of fatigue effects in the IMPL task. However, we think that this is unlikely, as the total time spent on the two tasks was ~10 min, and we did not find evidence of differences in performance across the first and second trials of the IMPL task. However, it is plausible that a group-level effect was ruled out because of other non-syntactic processing factors.

As argued in the introduction, observing that a sample of children does look (or does not look) at the target at above-chance levels would not provide clear support for Early Abstraction or Usage-Based accounts. We, therefore, compared models that assumed graded vs. discrete individual differences with the preferential looking data. The model assuming graded individual differences fit slightly better than the model assuming discrete individual differences, although this difference was smaller than two SEs and cannot, therefore, be distinguished from a sampling error. Moreover, we found no evidence supporting predictions about the sources of individual differences from either account. In the model of graded individual differences, neither the total number of verbs known nor lexical processing efficiency predicted overall looks to the target. In the model of discrete individual differences, lexical processing efficiency did not predict the proportion of looks to the target in the above-chance group, and the number of verbs known did not predict the probability that a given participant belonged to the above-chance group.

Given the ambiguous results above, we ran a set of exploratory analyses on our practice trials to determine whether our version of the IMPL task was, in principle, capable of capturing meaningful individual differences. We found moderate evidence to support this claim. The participants looked to the target action at above-chance levels once target preference was controlled for, although this effect was quite small when compared with previous studies. We found small but significant correlations between consecutive windows in the IMPL task, although not between consecutive trials, and we found that performance in the practice trials was related to the size of the verb vocabularies of the children, although not to their lexical processing efficiency. We encourage caution when interpreting these results, as our practice trials were not designed for the purpose of addressing these questions, and our analyses were exploratory. However, we believe that taken together, they suggest that our practice trials captured meaningful individual differences, although the size of these effects is smaller than would be desired.

This raises the question of why the practice and test trials differed in this regard, and what this means for using the IMPL to capture individual knowledge and variability. One possible explanation comes from the different patterns of correlations in the two phases of the task. While we observed no significant correlations between windows or trials in our test trials, we found significant correlations between windows, but not trials, in our practice data. Note that if the trials were consistently tapping the underlying knowledge of an individual, we should have seen some consistency in looking behaviors across trials and the windows within these trials, such that looking time behavior should be positively associated. We analyzed publicly available data from Messenger and Fisher (2018), whose data show some evidence of such consistency, although the behaviors of children still varied within and across experiments. We calculated the correlations between trials and time windows in Experiments 2 and 3, both of which tested the comprehension of novel verbs in passive sentences by children, with the only difference being that Experiment 3 was designed to reduce lexical processing demands. In Experiment 2, there was a significant correlation between trials 1 and 2 (*r* = 0.407), but not between windows 1 and 2 (*r* = 0.228). Experiment 3 yielded a different pattern of results, with a non-significant correlation between trials 1 and 2 (*r* = 0.265) and a significant correlation between windows 1 and 2 (*r* = 0.488). Thus, the study of Messenger and Fisher (2018) showed positive associations across trials and within windows, but the strength of the associations varied. In our data, we found some evidence for this in our practice trials, but not in our test trials. This variability suggests that the performance of children in the IMPL task is not always uniform within a given experiment, and does not predict individual trials in a consistent way across instantiations of this task. This points to the very likely possibility that the performance of the children within and across trials is historically contingent on prior looking behavior (i.e., from window to window, which is clear in all the eye-movement data, and from trial to trial, which is less often considered). However, the pattern of looking behavior and how it reflects the knowledge and/or learning during the task is difficult to ascertain and may be idiosyncratic in ways that do not only reflect linguistic knowledge. If this is the case, the average proportion of looks to the targets might contain enough relevant signals to measure individual differences in some instantiations, as in Messenger and Fisher (2018) and our practice trials, but not others, such as our test trials.

These results may be further evidence of the argument that tasks developed to perform well in experimental paradigms may not be suitable for individual difference studies (Hedge et al., 2018). In particular, because experimental designs seek to minimize between-subject variability, much of the variability in performance reflects error. This makes such tasks well suited for detecting group differences, but inadequate for modeling individual variability, for which meaningful between-subject variability is necessary (Hedge et al., 2018). This is increasingly becoming a concern in language acquisition research (Kidd et al., 2018a; Donnelly and Kidd, 2020). For example, in a recent study aimed at examining whether the comprehension of dynamic motion events by 10-month-old children was related to their vocabulary development, Durrant et al. (2020) found that looking times did not reliably capture individual differences. They point out that little is known about the drivers of the attention of children on these sorts of tasks; it may be that the comprehension of children is non-linearly related to looking time. Consistent with this suggestion, simulation, and empirical evidence suggests that children prefer videos of moderate complexity in looking time studies (Kidd et al., 2012; Piantadosi et al., 2014), looking away from the screen more often when images are of high or low complexity. While the relevant dependent measure of this study is notably different (proportion of total looks to one of the two stimuli, rather than overall time spent looking at the screen), it may be the case that, like in infant looking time studies, individual differences in the comprehension of children do not map to proportions in a monotonic manner.

The IMPL task has proven extremely useful for investigating differences in the linguistic knowledge between groups of children, thus earning its status as a workhorse in developmental psychology studies (e.g., see studies described in Golnikoff et al., 2015; Naigles, 2021). Many previous studies have found correlations between IMPL tasks and other linguistic and social variables, including our analyses of the practice trials, proving its utility as a measure of individual differences in some experimental contexts [Messenger and Fisher (2018); see Naigles (2021) for an overview of studies; although some studies have observed non-significant correlations between performance in the IMPL task and vocabulary, a finding often interpreted as evidence against Usage-Based accounts (Gertner et al., 2006; Scott et al., 2018)]. However, given the relatively unpredictable pattern of relationships between the trials described above, the precise form of the relationship between syntactic knowledge and the proportion of looks to the target is unclear. This poses a challenge to complicated models of individual differences, such as those reported here. Future studies should aim to understand how individual differences in the comprehension of children map onto IMPL tasks. A promising tool for doing so is the combination of cognitive process models and psychometric models, so-called cognitive psychometrics (Voorspoels et al., 2018). Such studies may prove a necessary pre-requisite for testing the more precise predictions of individual differences discussed in this report.

## Conclusion

In this study, we tested the competing predictions of the Early Abstraction and Usage-Based accounts of early grammar by considering the nature and structure of individual differences in the comprehension of English transitive sentences containing novel verbs by 2-year-old children. Overall, we found little evidence favoring either a set of predictions about the structure or the source of individual differences. However, the interpretation of our results was complicated by the low correlation between trials and the consistent preferences for particular actions. While we believe that our approach to modeling individual differences holds much promise for adjudicating between theoretical debates in language acquisition, more work on the psychometric properties of commonly used experimental methods, such as the IMPL, is necessary to precisely quantify the varying abilities of children. Thus, despite our unclear pattern of results, we see significant merit in pursuing the mapping of individual differences in development, although there is much more theoretical and methodological work to do (see Kidd and Donnelly, 2020).

## Data Availability Statement

The data are available on the osf page cited in the article https://osf.io/tqz8b/.

## Ethics Statement

The studies involving human participants were reviewed and approved by Australian National University Human Research Ethics Committee. Written informed consent to participate in this study was provided by the participants' legal guardian/next of kin.

## Author Contributions

All authors listed have made a substantial, direct and intellectual contribution to the work, and approved it for publication.

## Funding

This research was supported by the Australian Research Council (CE140100041: CI Kidd).

## Conflict of Interest

The authors declare that the research was conducted in the absence of any commercial or financial relationships that could be construed as a potential conflict of interest.

## Publisher's Note

All claims expressed in this article are solely those of the authors and do not necessarily represent those of their affiliated organizations, or those of the publisher, the editors and the reviewers. Any product that may be evaluated in this article, or claim that may be made by its manufacturer, is not guaranteed or endorsed by the publisher.

## Footnotes

^1^It is important to emphasize that there is a wide variety of Early Abstraction accounts, and we have only summarized those most typically invoked when explaining findings from the IMPL task. It is possible that other versions of the Early Abstraction account would make different predictions.

^2^As with our discussion of the predictions from the Early Abstraction accounts, we emphasize that there are potentially other Usage-Based accounts, not typically referred to in research on this task, that could make different predictions.

^3^This is equal to (Y^*^(N−1) + $\frac{1}{2}$)/N, where Y is the raw proportion and N is the number of eye-tracking samples using which Y was calculated.

^4^Sufficient LWL data were available for 88 of the 92 participants.

^5^Sufficient vocabulary data was available for 88 of the 92 participants.
